# Reliability of Greek version of the Toronto empathy questionnaire in medical students and associations with sociodemographic and lifestyle factors

**DOI:** 10.1186/s40359-022-00824-6

**Published:** 2022-05-02

**Authors:** Polychronis Voultsos, Fotios Chatzinikolaou, Angeliki Papana, Aspasia Deliligka

**Affiliations:** 1 Laboratory of Forensic Medicine and Toxicology (Medical Law and Ethics), School of Medicine, Faculty of Health Sciences, Aristotle University, University Campus, 541 24 Thessaloniki, Greece; 2 Department of Economics, School of Economics and Regional Studies, University of Macedonia, Egnatia Str 156, 546 36 Thessaloniki, Greece; 3 AHEPA University Hospital, Kiriakidi Str 1, 546 21 Thessaloniki, Greece

**Keywords:** Empathy, Toronto composite empathy scale (TCES), Medical students, Greek/Greece, Validation

## Abstract

**Background:**

Empathy is an important key driver of any therapeutic relationship. It is beneficial for both physicians and patients. Enhancing physician’s empathy should be an important goal of medical education. As there was a literature gap regarding the topic of empathy among medical students in Greece, this study aimed to contribute to filling this gap.

**Methods:**

A cross-sectional study was conducted. A socio-demographic questionnaire and the 52-item Greek version of the Toronto composite empathy scale (TCES) for measuring the cognitive and emotional aspects of empathy in both personal and professional life was administered to all the medical students in the Aristotle University of Thessaloniki, in Greece. Descriptive statistics were displayed for demographics. The associations of the variables were quantified by Chi-2 independence tests and Pearson’s Correlation Coefficient. The reliability and validity of the questionnaire was determined by Cronbach’s α, Hotelling's T-Squared Test, and Pearson correlation. Paired and Independent Sample T-Tests and One-way ANOVAs indicated statistically significant mean differences among the variables or subgroups of the variables.

**Results:**

The 52‐item TCES, 26 for the personal (Per) setting and another 26 for professional (Pro) life, equally divided into cognitive (Cog) and emotional (Emo) empathy in each case. The overall reliability of the TCES questionnaire was found to be high (Cronbach's α = 0.895, significant positive correlations between the subscales). The mean total score of empathy showed that students had a moderately high empathy. Further, there was a statistically significant difference in means between the Per-Cog and Per-Emo settings (*p* < 0.001), the Pro-Cog and Pro-Emo (*p* < 0.001), the Per-Cog and Pro-Cog (*p* = 0.004), and the Per-Emo and Pro-Emo (*p* < 0.001). Females had significantly higher empathy scores (mean score 208.04) than males (192.5) on the Per-Cog, Per-Emo and Pro-Emo subscales. Furthermore, a positive correlation was found between empathy and factors such as love for animals, interest in medical ethics, belief in God, having an ill person in the family, class year or carrier intention.

**Conclusions:**

The TCES is applicable to medical students. For the most part our findings were consistent with previous literature. However, we identified some nuances that might draw researchers’ attention. The results of this study may contribute to plan interventions in the curriculum to enhance empathy in the medical students.

**Supplementary Information:**

The online version contains supplementary material available at 10.1186/s40359-022-00824-6.

## Background

### What is empathy in the context of health care

Empathy is difficult to define and constitutes a controversial concept with different components. It is a multilayered phenomenon [1]. In the context of health care and medical education, it is defined as the capacity of understanding of the patient’s situation, inner experiences, feelings, concerns and perspectives (view the outside world from the other person’s perspective), combined with a capacity to communicate that understanding and act helpfully on that understanding [2–6]. However, an empathetic physician should not engage with patients’ feelings, emotions and perspectives [7]. Larson and Yao [8] put it best in saying “clinical empathy has been compared to an actor’s skill at engaging in and reacting to others’ emotions”. Joining the feeling of the patient is sympathy [9]. Empathy and sympathy have different neurophysiological background [10]. Evidence from neuroscience conceptualises empathy as predominantly an intellectual response involving the neocortex of the brain, and sympathy as predominantly an emotional response involving the limbic system of the brain [11]. Emotional regulation is a key component to an appropriate empathic response [12]. Lack of emotional regulation limits physician’s cognitive resources which should be available to cure his or her patient [13]. Better emotional regulation leads to more functional affective empathy [4].

Recently, it has been argued that there is a positive association between self-esteem and empathy [14], and that enhancing students’ ability to introspect (self-awareness) is crucial to enhance their empathy [4].

In the context of health care (at least), empathy is considered a complex and multidimensional concept that has cognitive, emotional, moral, and behavioural dimensions [15–17].

In psychology, new empathy models have been developed to capture the essence of empathy, such as the mirror-neuron theories [18] and perception–action model (PAM) [19]. Preston and de Waal argue that empathy is a deeply personalized phenomenon, and any model that fails to address this does not capture its essence. The authors argue that perception–action model (PAM) is a significantly broader concept that encompasses the mirror mechanism but also “takes into account the deeply personal way that empathy depends on personal experience”, thus providing a deeper explanation of empathy [20].

### The distinction between cognitive and affective component of empathy

Some authors consider that cognitive empathy is distinguished from affective empathy [21]. Furthermore, it is argued that the cognitive component is the most prevalent, whereas the affective is the least [22, 23]. Several studies used a definition for empathy proposed by Hojat and LaNoue [24], which regards empathy as a predominantly cognitive attribute [25]. Gladstein who conceptualizes empathy as a two‐dimensional model (cognitive and affective dimension), has long before simply and clearly described these dimensions. The author states that the cognitive component refers to ‘intellectually taking the role or perspective of another person’, whereas the affective component consists of ‘responding with the same emotion to another person’s emotion’ [26]. Preston and de Waal [27] consider a clear distinction between emotional and metacognitive empathy. Emotional empathy may be an automatic process [28] while meta-cognitive empathy is an effortful perspective-taking which is considered significant in the clinical context [29]. Effortful perspective-taking decreases personal distress and increases empathetic concern [30]. Hojat and colleagues portrayed ‘perspective-taking’ as a core ingredient of empathy (especially in the patient-physician relationship), which however, can hardly be distinguished from ‘standing in the patients’ shoes’ (a second ingredient). The third ingredient is ‘compassionate care’ [2, 24]. It is noteworthy that Hojat et al. suggest that “physician empathy is a multidimensional concept involving at least three components. The most important component is perspective taking, an outcome consistent with that reported for the general population. Other components of empathy are compassionate care and standing in the patient’s shoes, which are both specific to the patient-physician relationship” [2]*.* Hojat et al. define empathy as “a cognitive (as opposed to affective) attribute that involves an understanding of the inner experiences and perspectives of the patient, combined with a capability to communicate this understanding to the patient” [9]. Furthermore, the authors state, “The key feature of empathy, according to our definition, is understanding, rather than affective involvement with patients' experiences. The affective domain is a key component of sympathy, rather than empathy” [9]. Other authors state that “clinical empathy—the compassionate professionalism of a skilled clinician—is affective and metacognitive, contextual and interpersonal, and difficult to assess” [27, 29]. “While it relies on general empathy—the compassion and understanding in everyday life—it also requires a degree of emotional detachment and objectivity” [29]. Perspective-taking “is similar to, but clearly not the same as, cognitive empathy” [31]. A great part of literature emphasizes cognitive empathy as most important to the relationship between physician and patient. However, it is arguably stated that “this conceptualization is both simplistic and misleading” [32]. It is true that the distinction between cognitive and affective component of empathy is relevant. For instance, it is argued that “the cognitive dimension seems more receptive to a training program, while the affective component would be more innate” [33]. However, “*these components* are intimately linked and interdependent” [34, 35]. The cognitive and affective component of empathy are interwoven. de Waal and Preston state: “Recent research on empathy in humans and other mammals seeks to dissociate emotional and cognitive empathy. These forms, however, remain interconnected in evolution, across species and at the level of neural mechanisms” [19].

There is convincing empirical evidence from developmental science, social neuroscience, and clinical neuroscience that the cognitive and affective facets of empathy interact in the experience of empathy” [32]. Ponnamperuma et al. [36] put it best in saying “notwithstanding the cognitive–affective dichotomy, most studies view empathy as a holistic construct, while acknowledging its different dimensions”.

### The role of empathy in clinical practice

There is evidence suggesting that a physician’s empathy not only plays a central role in the physician–patient relationship (promotes the patient-centered communication which stands at the very heart of medicine), but also positively affects the patient’s satisfaction as well as the therapeutic outcomes [2, 36–45]. Empathy is strongly related to the humanitarian aspect of medicine. Patient-oriented care is at the core of holistic care. Students that participated in qualitative research considered that empathy is a virtue that makes you a ‘better’, more selfless person [46].

Hojat concludes “that empathic engagement in the health care and human services is beneficial not only to the patients, but also to physicians, other health care providers, administrators, managers, health care institutions, and the public at large” [47].

Hojat et al. and Steinhausen et al. emphasize the relationship between physician’s empathy and trauma surgery patients and diabetic patients, respectively [39, 48].

There is evidence that empathetic physicians have higher levels of clinical competence [49] and reduced number of legal claims against them [6]. In that connection, the Association of American Medical Colleges (1998) stated that “physicians must be compassionate and empathetic in caring for patients and must be trustworthy and truthful in all of their professional dealings”. “Anyhow, all authors agree to say that empathy is important” [4]. At any rate, empathy motivates prosocial behavior. Note, however, that this motivation is context—dependent [50].

### “Teaching” medical students to be empathetic

Enhancing physician’s empathy should be an important goal of medical education [6, 17, 23, 51]. There is “a need to incorporate a regular training program into the existing medical curriculum, to enhance empathy and prevent its decline over the years” [52]. Multi-institutional international research would be most helpful for this to be done [53].

Literature has raised the question whether empathy is a teachable (acquired) skill or a hereditary trait. Indeed, both cognitive and affective empathy are said to be associated with hormones (dopamine/testosterone and oxytocin/arginine-vasopressin/serotonin, respectively) [10]. Moreover, it is argued that specific gene’s function may be associated to empathy. It is suggested a significant association between the rs53576 OXTR gene polymorphism and empathy (particularly the affective aspect of empathy), especially among women [54]. Note, however, that environmental influences may play a key role to the expression of these particular genes. Kataoka et al. [55] concluded that “targeted educational programs to enhance empathy in medical students can have a significant effect”. Many studies suggest that undergraduate studies should include a range of intervention strategies to enhance students’ empathy of medical students [56] or dental students [57]. As presented below ("Discussion" section), research has shown a striking decline in empathy over the years as medical studies progress. Dehning et al. [58] found very low empathy among first-year medical students and highlights the need for “inclusion of specific training in cognitive and emotional empathy in medical education”. Ye et al. [59] highlight that “empathy-focused training during early clinical contact can improve the empathetic capacity of undergraduate medical students”.

Hojat provided “10 approaches for enhancing empathy in medical education: improving interpersonal skills, audio- or video-taping of encounters with patients, exposure to role models, role playing (aging game), shadowing a patient (patient navigator), hospitalization experiences, studying literature and the arts, improving narrative skills, theatrical performances, and the Balint method” [47]. Note, however, that some authors suggested that the empathetic skills enhanced through these interventions’ strategies (which may significantly increase immediately after an intervention) may degrade over time [60, 61].

Already, in many countries medical schools have attempted to develop strategies to enhance empathy in their medical students [25]. Even if there is intrinsic motivation and self-efficacy, a supportive learning environment in required to develop students’ effective clinical empathy in various clinical contexts [25]. The General Medical Council [62, 63] as well as the Association of American Medical Colleges [64, 65] have emphasized the need for teaching empathy as a professional skill and competency. Importantly, humanities curriculum (“an extensive 3-year preclinical medical humanities curriculum”) may prevent the empathy decline [6]. Olsen and Gebremariam [66] found that medical students “who majored in humanities or interpretive social sciences disciplines have higher empathy scores than their peers who majored in the positivistic social sciences and STEM (science, technology, engineering, and mathematics) disciplines”.

Note, however, that theoretical education is necessary though not sufficient to enhance students’ empathy. Santiago et al. [67] found medical student's “empathy levels being higher when earlier and more intense contact with patients accompanied by skilled tutors was developed”. It arguable that “hands-on approaches to diagnosis and treatment, and patient-centered care” may play a crucial role in enhancing medical students’ empathy [68]. Rivas put it best in saying: “First, academic workshops and group forums, supervised by doctors with bioethics training, can introduce empathy and related topics for discussion. Second, clinical rotations can help students to gain additional insights through interactions with patients and learning about their real‐life experiences as patients in the health care system” [69]. It is arguably highlighted the role of the contact between medical students and patients in enhancing their empathy. Interestingly, Davison and Lindqvist [70] strongly suggest the benefits of medical students working as health care assistants.

It is crucial to bear in mind that “while students may understand the importance of empathy, there is currently no consensus on the appropriate method for teaching this quality” [71]. For instance, a widely used method of enhancing medical students’ empathy is the role-play and “the participation in both sides of the doctor–patient partnership” [72]. At any rate, it is crucial to bear in mind that “the current empathy intervention literature is limited by a variety of methodological weaknesses” [60].

At any rate, the crucial question is whether empathy “is a ‘nature’ or ‘nurture’ phenomenon or a combination of both”. It remains unclear [49]. It has been argued that empathy is an innate attribute and early upbringing-driven capacity, which can be enhanced by learning [73].

Empathy in medical education is not a “virgin” topic in research. However, there are still many gaps to be filled. In Greece, limited research has been conducted on this topic. This study aimed to contribute to filling this gap. To our knowledge there is little research measuring empathy among medical students in Greece. The answers to the research questions for this study could provide a psychometrically sound instrument that might be used in future research on the factors that affect the level of empathy among medical students in Greece. Moreover, in the context of previous literature, there was a need for researchers to further explore the psychometric properties of the Toronto Composite Empathy Scale (TCES) [74] (and more particularly the Greek version of the TCES) among medical students.

The study instrument was developed to include an introductory cover letter, a number of questions created to assess the demographic characteristics of study participants (first part of the questionnaire), and the Greek version of the Toronto Composite Empathy Scale (TCES) (second part of the questionnaire). The survey instrument contained 20 demographics questions, such as age, gender, financial status of family, siblings, the specialty students intend to follow. The second part of the questionnaire consisted of 52 items from the Greek version of the Toronto Composite Empathy Scale (TCES), 26 for the personal (Per) setting and another 26 for professional (Pro) life, equally divided into cognitive (Cog) and emotional (Emo) empathy in each case. Therefore, we examined the four thirteen-item subscales (Per-Emo, Per-Cog, Pro-Emo, Pro-Cog) and the overall empathy scale. A six-point Likert scale with no neutral position was used in the analysis. Items are scored as follows: at no time = 1, most of the time = 2, slightly less than half times = 3, slightly more than half times = 4, some of the time = 5, all the time = 6. The Greek version of the TCES has previously been validated among dental students [74]. We wondered whether it might also be applicable to medical students. As the Greek version of the TCES demonstrated acceptable validity and reliability among dental students, we hypothesized that in all likelihood it also might be validated in the environment of medical students provided that the items of the questionnaire were not specific for dental students.

Furthermore, the results of this study are expected to contribute to increase the level of empathy in medical students during the course of the study by reconstructing the medical curriculum. The results of this study may contribute to design appropriate educational strategies and interventions in the curriculum to enhance empathy in our medical students. It should be noted that over the recent years the Medical School of the Aristotle University of Thessaloniki began showing an interest in integrating communication skills and empathy programs for the students in the school curriculum (that is currently under reconstruction).

To our knowledge, the TCES has not been widely used in research. More specifically, it has not been used for medical students in Greece or elsewhere. A study published in a Chilian journal found that the Spanish version of the TCES demonstrated acceptable validity and reliability and could be used to assess personal and professional empathy in Chilean dental students [75]. Tsiantou et al. [74] examined the psychometrics of the Greek version of the TCES in Greek dental students. The authors found that the Greek version of the TCES has acceptable or good internal consistency for all subscales (Cronbach’s alphas were “very similar to those reported by Yarascavitch et al.”) [74]. They state that their findings suggested good convergent validity, good discriminant validity and good test–retest reliability for the scale [74]. In the Greek context Katsari et al. [76] used the TCES in their study aiming to estimate physicians' self-assessed empathy. The authors administered a composite questionnaire (in which the TCES was included) to the participants in their study [76]. Moreover, a study published in a Greek journal used TCES to evaluate empathy among nurses in a public hospital in the Athens broader region [77].

### Research questions

The primary research question that defined the focus of this study was as follows:

Is the Greek version of the Toronto Composite Empathy Scale (TCES) reliable to medical students?

The secondary research questions were as follows:

What are the empathy levels among medical students at the Aristotle University of Thessaloniki (Greece)?

How are some lifestyle or socio-demographic factors correlated with empathy among medical students at the Aristotle University of Thessaloniki (Greece)?

## Materials and methods

### Procedure/study setting

This type of research design is a cross-sectional study. The online survey was conducted during the months of February and May 2021. More specifically, a questionnaire was distributed via Google Forms to students enrolled in the School of Medicine of the Aristotle University of Thessaloniki, the second largest medical school in Greece. The entire duration of undergraduate medical degree in Greece is 6 years. The undergraduate curriculum of the School of Medicine of the Aristotle University of Thessaloniki did not address the topic of empathy, at the time of the interviews.

### Participants

The study involved stratified random sampling. Our target population consisted of undergraduate medical students enrolled in the Medical School of the Aristotle University of Thessaloniki, during the 2020–2021 academic year. The participants were randomly selected from different academic years (freshman to senior). All potential participants were invited to fill out an anonymous online self-report questionnaire. The study instrument was electronically distributed and collected online via Google forms and using learning platforms, student information systems and university websites. Given that two authors of this study (PV and FC) are associate professors at Aristotle University of Thessaloniki Medical School, we had access to these university websites or platforms. A reminder email was sent to them 2 months after the distribution. Responses were collected for three months.

The sample size calculator “The Survey System” was used for calculating a minimal sample size [78]. Moreover, the standard formula: n = P × (1 − P) × z^2^/d^2^ was used for confirming the minimal sample size [78]. Given that during the last decade or so, approximately 150 students were enrolled in the Medical School of the Aristotle University of Thessaloniki in each academic year, we determined the sample size based on a known total population, the most conservative percentage (*p* = 50%), and a confidence level of 95%. The confidence interval (margin of error) that was used for determining the sample size was 5.68.

The survey was conducted in line with the principles of the Declaration of Helsinki and was approved by the Ethics Review Committee of the Medical School of the Aristotle University of Thessaloniki. In the introductory cover letter, the students (potential participants) were informed of the study goals and that their participation is voluntary. The students were assured that their participation “is and will remain anonymous”. All participants provided written consent to participate in the study online. All participants were aged > 18 years. Ultimately, in this study 96.4% of the participants declared that became aware of the nature and purpose of the research or/and on the collection, processing, and storage of the data, while the remaining 3.6% were missing values.

### Measures

More precisely, prior to the development of the Jefferson Scale of Empathy (JSE) none psychometrically sound instrument was specific enough to meet the need for an operational measure of empathy in the context of medical students and physicians. In the year 2002, Hojat et al. [9] developed the JSE in response to this demand. The JSE was developed to measure cognitive rather than affective or emotional empathy. Since then, and until today, the JSE is widely used to measure empathy among medical students in many studies. In the year 2009, Yarascavitch et al. [79] developed the Toronto Composite Empathy Scale (TCES) to measure the cognitive and emotional aspects of empathy, in both personal as well as professional settings, in dental students. This scale attempted to formulate a “consensus” among four scales related to empathy. The TCES is a combination of questions from four scales: JSPE-HP that consists of 20 questions and has been used to measure empathy in health care professionals, and three empathy-related scales: the Interpersonal Reactivity Index-IRI, the E-Scale and the short form of the Empathy Quotient-EQ [79]. The TCES consists of 52 questions, 26 for the professional aspect of empathy and another 26 for the personal aspect of empathy, equally divided into cognitive and emotional empathy in each case. The fifty-two questions of the TCES are divided into four subscales: Personal Emotional, Professional Emotional, Personal Cognitive, and Professional Cognitive, each consisting of 13 questions. Every subscale's score ranges from 13 to 78, with higher score denoting higher level of empathy [76, 79]. Yarascavitch et al. found acceptable internal consistency for the TCES. The Cronbach's alpha values were as follows: 0.759 for the Personal Cognitive subscale, 0.765 for the Personal Emotional, 0.814 for the Professional Cognitive and 0.768 for the Professional Emotional. Since then, there have been scarce studies using the TCES [79].

Finally, it should be noted that possible scores for *each subscale* range from 13 to 78. Higher scores indicate higher levels of empathy.

### Statistical analysis

Descriptive statistics were used to summarize the demographic characteristics of the participants, such as frequency distributions and percentages. Regarding the TCES items, the subscales and total scores, means and standard deviations are displayed. The associations between the demographics were reported in terms of the chi-2 independence tests. The internal consistency of the subscales and of the TCES questionnaire was analyzed with Cronbach’s α and Hotelling's T-Squared Test, while Pearson correlation was utilized for the examination of the validity of the questionnaire. Pearson’s Correlation Coefficient was estimated to investigate possible relationships between the individual items. The mean differences between the subscales were investigated based on Paired Samples t-tests. Significant differences between the subscales and TCES from subgroups on the demographic characteristics were established using Independent Sample T-Tests and One-way ANOVAs.

The analysis was performed using the statistical program SPSS 25.0 (Statistical Package for the Social Sciences) of IBM.

## Results

### Demographics

A total of 224 medical students participated in this study, whereas 65.2% were women and 33.9% men. Most of the participants were up to 25 years old (91.1%), 7.6% were up to 35 years old and the remaining 1.3% were over 35 years old. All participants were above 16 years of age. Almost half of them were born in a big city (48.7%), 37.1% in a provincial town, 7.6% in a town and 4.9% in a village. 37.5% of the participants grew up in a big city, 35.7% in a provincial town, 14.7% in a town, and 12.1% in a village 89.7% of them were born and 90.6% were raised in Greece. 71.9% of the participants were living together with other people. 74.6% never had going through a serious adventure with his/her health.

Regarding the family they were raised, 58.9% replied that were raised in a large family. A high percentage of the participants had siblings (92%) and did not have children (97.8%). Most of them were raised in a house without grandparents or elderly people (77.2%). In their family or in their immediate environment there was a person who was seriously ill with a percentage of 46%. Their families mainly did not have enough financial comfort nor financial difficulties (44.6%), some had enough financial comfort (29.9%) and even fewer went through difficult phases financially (21.9%). At least one parent of the participants graduated from the university (78.6%).

According to their love to animals, over half of them loved animals very much (53.6%), quite loved them (33.5%), very little loved animals (8%), did not care about animals (4%) and 0.9% hated them.

The highest participation was from students in their 1rst year of studies, followed by 3rd years students (18.3%), 2nd years students (16.5%), 6th year (13.4%), 4th year (12.5%), 5th year (10.7%) and finally on their degree (2.2%). Regarding the specialty they want to follow, the highest percentages were for General Surgery (9.8%), “did not know” (9.4%), Psychiatry (7.6%), Pediatrics (7.1%). Almost half of them had not decided on their future career (42.9%), 25.4% intended to work on public sector, 20.1% in private sector and 11.6% intended to follow an academic career. Finally, 46.9% were moderately interested in being informed often about medical ethics issues, 37.1% were very interested, 14.3% were a little interested and 1.3% were not interested at all.

Religious were the 62.1% of them, 21% did not believe in God and the remaining did not know or wanted to answer this question.

### TCES items

The TCES questionnaire has been replied by 224 students. Missing values for Personal setting varied from 0 to 3, while an increased number was obtained for Professional setting reaching out 40 missing replies. A significant negative correlation was found between the number of missing replies of each participant and the study year (Pearson = -0.336, *p* < 0.000), with students from 1rst and 2nd years mainly not responding to most items.

Table 1 displays the descriptive statistics for TCES items with personal setting. The highest values, indicating more empathy, were revealed for items Q9 ^R^, Q35 ^R^, Q26 ^R^, while the minimum values (less empathy) were revealed for items Q50, Q36, Q10. Mainly positive correlations were observed between the TCES items, with strongest ones for Q7 ^R^–Q33 ^R^ (0.724), Q10–Q36 (0.651), Q14–Q40 (0.633) and Q38–Q47 (0.613).

**Table 1:** Descriptive statistics for subgroup scales with personal setting

Item	Cognitive		Item	Emotional	
	Mean	SD		Mean	SD
Q1	4.12	1.262	Q5	3.71	1.489
Q2	4.58	1.289	Q7 ^R^	2.84	1.178
Q3 ^R^	4.75	1.030	Q10	2.13	1.131
Q4	4.33	1.273	Q12	3.48	1.539
Q6	4.77	0.938	Q13	2.75	1.325
Q8 ^R^	4.03	1.287	Q15 ^R^	3.28	1.448
Q9 ^R^	5.25	0.935	Q16	3.44	1.262
Q11	3.23	3.48	Q18	4.61	1.193
Q14	4.12	1.268	Q20	3.83	1.443
Q17	4.81	0.910	Q21	3.17	1.450
Q19	4.11	1.335	Q24	3.23	1.286
Q22	4.81	0.949	Q25 ^R^	4.29	1.318
Q23 ^R^	4.28	1.176	Q26 ^R^	4.95	1.121

Items with reversed scores are noted with superscript ^R^

Table 2 displays the descriptive statistics for TCES items with professional setting.

**Table 2:** Descriptive statistics for TCES items with professional setting

	Cognitive			Emotional	
	Mean	SD		Mean	SD
Q27	4.15	1.243	Q31	2.56	1.217
Q28	4.75	1.084	Q33 ^R^	2.71	1.134
Q29 ^R^	4.93	0.979	Q36	2.12	1.170
Q30	4.39	0.979	Q38	3.49	1.607
Q32	4.64	0.993	Q39	2.65	1.286
Q34 ^R^	4.31	1.231	Q41 ^R^	2.53	1.161
Q35 ^R^	5.24	0.997	Q42	2.61	1.190
Q37	3.70	1.336	Q44	4.87	1.168
Q40	4.35	1.271	Q46	3.51	1.457
Q43	4.60	1.044	Q47	3.28	1.579
Q45	3.64	1.435	Q50	2.02	1.075
Q48	4.87	1.007	Q51^R^	4.28	1.307
Q49 ^R^	4.56	1.042	Q52^R^	4.81	1.258

Items with reversed scores are noted with superscript ^R^

### TCES subscales

As previously discussed, missing answers were mainly from the Professional setting. Therefore, 215 and 216 valid cases were considered for the Per-Cog and Per-Emo settings, respectively, while for the Pro-Cog and Pro-Emo only 43 valid cases were found.

First, the internal consistency of each subscale has been established on Cronbach’s α, which indicated that the set of items of each subscale were closely related as a group. Specifically, α = 0.748 for Per-Cog, α = 0.806 for Per-Emo, α = 0.830 for Pro-Cog, α = 0.805 for Pro-Emo. Hotelling’s T-Squared Test further confirmed that all items on the scale had the same mean (*p* < 0.001 for all subgroups).

Possible scores for each of the four subscales (Per-Cog, Per-Emo, Pro-Cog, Pro-Emo) ranged from 13 to 78, where higher scores refered to greater levels of empathy. The mean score (and standard deviation, SD) on the Per-Cog was 57.14 (SD = 7.49), on the Per-Emo was 45.73 (SD = 8.00), on the Pro-Cog 58.17 (8.53) and on the Pro-Emo was 41.50 (9.20), respectively.

Paired Samples T-Tests showed that there was a statistically significant difference in means between the Per-Cog and Per-Emo settings (*p* < 0.001), the Pro-Cog and Pro-Emo (*p* < 0.001), the Per-Cog and Pro-Cog (*p* = 0.004), and the Per-Emo and Pro-Emo (*p* < 0.001).

**Fig. 1:**
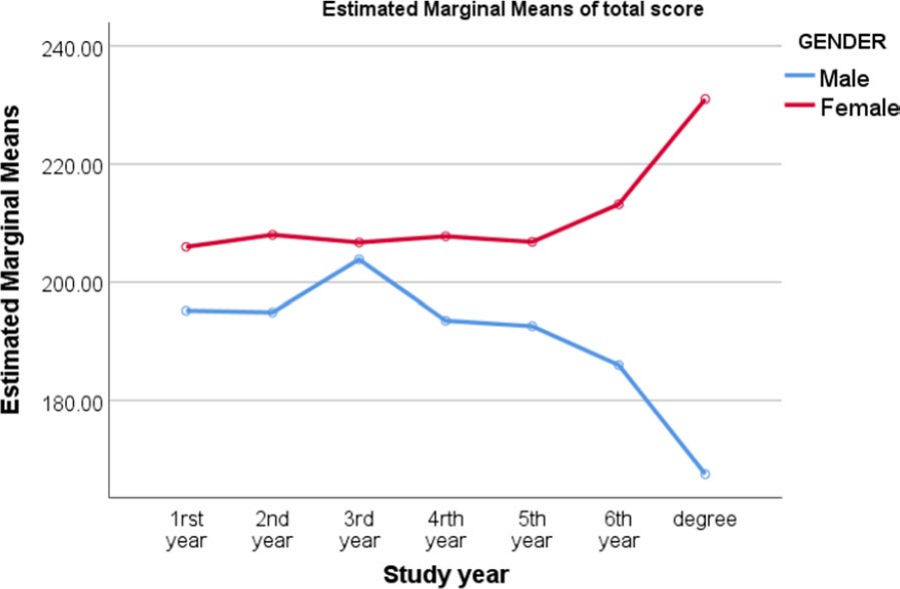
Profile plot from two-way ANOVA

Females had significantly higher empathy scores on the Per-Cognitive, Per-Emo and Pro-Emo subscales (Table 3). It is noticeable that this cross-sectional study in one medical school in Greece showed that females did not demonstrate statistically significant decline in empathy total score over the course of medical school, with males demonstrating a statistically significant decline in empathy as they began their clinical training (in the fourth year of studies). Interestingly, the empathy measures of senior year female students were higher than the scores of female freshmen (see Fig. 1). A longitudinal cohort study is needed to better measure variations in students' empathy scores throughout medical school.

**Table 3:** Means and standard deviations (SD) of the Greek version of TCES by gender, and *p*. from Independent Sample T-test for equality of means

Subscale	Males mean (SD)	Females mean (SD)	*p*
Per-Cog	54.73	58.4	0.001*
Per-Emo	42.79	47.31	0.000*
Pro-Cog	57.48	58.71	0.353
Pro-Emo	36.97	43.80	0.000*

Statistically significant differences are noted by *

Furthermore, as regards the effect of gender and years of studies on total score, a Two-way ANOVA was conducted that examined the effect of gender and years of studies on total score. An interaction between gender and years of studies could not be demonstrated (*p* = 0.990). Only gender seemed to have a main effect on total score. Adjusted r squared suggested that 5.7%% of the variance in total score was attributable to gender and years of studies (Additional file 1: Table S1).

The Tests of Between-Subjects Effects Table (Additional file 1: Table S2) indicated whether either of the two independent factors or their interaction were statistically significant. Results suggested that only the factor GENDER was statistically significant (*p* = 0.000), while the factor years of studies (*p* = 0.990) and the interaction variable of the two factors were not (see Fig, 1).

Additional statistically significant mean differences found based on Independent T-Test or One-Way ANOVAs (for more than 2 subgroups) for different subgroups of demographic characteristics are described in Additional file 1: Table S3. Important factors that differentiate results were having an ill person in the family, loving animals, year of studies, career intention, religion and interest on ethical issues in medicine.

### TCES

The overall reliability analysis of the TCES questionnaire was high (Cronbach's α = 0.895, *p* from Hotelling’s T-Squared Test < 0.000). The total score was extracted from 172 cases (76.8% of total sample). Possible total scores from the 52 items of the TCES ranged from 52 to 312. The mean total score of empathy was 203.16 (SD = 24.089) which shows that students had a moderately high empathy. An increased total mean score was estimated for females. One-Way ANOVA tests showed that the loving animals, believing in a God and the interest in medical ethics played an important role in the total mean score of the corresponding subgroups (see Additional file 1: Table S4).

As regards the statistical difference in mean total score of two specialty categories, A (people-oriented specialties) and T (technology-oriented specialties), there was no significant difference in the mean total score of students that aimed to choose technology-oriented specialty (mean total score 205.6) and people-oriented specialty (mean total score 205.96). There was also no significant difference in the mean score of the subscales for the demographic characteristic of specialty category (Additional file 1: Table S5).

Note, however, that subgroups of 17 demographics did not have statistically significant total mean score differences (see Additional file 1: Table S6).

Ultimately, the Pearson correlations between the four subscales have been estimated. Regarding convergent validity, significant positive correlation was estimated between the two cognitive subscales (Pearson = 0.745, *p* < 0.001) and the two emotional subscales (Pearson = 0.802, *p* < 0.001). Concerning discriminant validity, significant positive correlation was found between the two personal subscales (Pearson = 0.243, *p* < 0.001). There was however no significant correlation between the two professional subscales (Pearson = 0.128, *p* < 0.089). Further evidence was provided by examining the item intercorrelations for all item pairings for each subscale, i.e., by examining convergent and discriminant validity. A high percentage of significant and positive correlations was found considering all pairs of items of each subscale, i.e., 64.1% regarding items of Per-Cog subscale, 85.9% of Per-Emo, 79.49% of Pro-Cog and 74.36% of Pro-Emo. To establish discriminant validity, the relationships between items from different subscales were estimated. Results suggested that the majority of items from different subscales were not significantly correlated. For example, 76.9% of the correlations between item Q1 (from Per-Cog subscale) and items from Per-Emo subscale were not significant.

## Discussion

The preliminary validation of the Greek version of the Toronto Composite Empathy Scale (TCES) demonstrated good validity and reliability (Cronbach's α = 0.895, *p*. from Hotelling’s T-Squared Test < 0.000) among medical students and could be further used in case of samples of medical students. The students that participated in our study had a moderately high empathy. As presented below, this is not surprising when measuring empathy among medical students, especially in a Western-oriented country, and given that altruism may be an important motivation for medical students to study medicine. Results also suggested that there was a statistically significant difference in means between the two personal subscales and between the two professional subscales, as well as between the two cognitive subscales and the two emotional subscales. The professional subscales showed lower levels of empathy than the personal ones. The emotional subscales showed lower levels of empathy than the cognitive ones, with the lowest levels of empathy being observed on the professional emotional empathy subscale.

We must provide some discussion about the statistically significant difference in means between the subscales of TCES. As suggested by our findings, the medical students demonstrated different levels of cognitive and emotional empathy in both personal and clinical settings. Emotional empathy was lower than cognitive empathy, with professional emotional empathy being the lowest. Several factors may account for this phenomenon. We attempted to provide a potential explanation for it. There were some common factors that may negatively affect emotional empathy in medical students, with some of these factors negatively affecting the professional aspect of emotional empathy. First, the participants in this study could probably be aware of the fact that expressing your emotions via clinical empathy is a stressful habit that can lead to chronic weariness [80]. This might make them unwittingly want to disengage themselves from emotionally cumbersome situations and may be one potential reason why emotional empathy was lower than cognitive empathy. Second, the medical students who participated in this study were required to express their own understanding of empathy in a healthcare context of changing professional dynamics where, however, traditionally the medical profession downplays the expression of emotion [81]. This may be one potential reason why the professional empathy showed the lower score than personal empathy. In that regard, we can hypothesize that while many medical students were naturally empathetic persons with great empathy in their personal everyday life, when they envisioned themselves working in the clinical context, they tended to suppress their empathy. Third, emotional engagement is a core element of professional empathy in the clinical context. Vinson and Underman [80] have recently theorized clinical empathy as “emotional engagement in the contemporary clinical encounter”. The authors described “the consistent performance of clinical empathy as a form of emotional labor”, and more precisely, a “contextually specific set of emotional labor techniques” [80]. Furthermore, emotional intelligence has been found to be related to more empathetic attitudes among medical students [82]. This may be one potential reason why the professional aspect of emotional empathy showed the lowest score. At any rate, as the participants in this study never had a training program for communication skills and empathy, they might be at a loss to have an in-depth view of clinical empathy. As clinical empathy is an essential quality and matters a lot for clinicians, further research on the topic ‘emotional empathy in undergraduate medical education’ is required. The Toronto Composite Empathy Scale (TCES) is a psychometrically sound instrument for measuring the cognitive and emotional aspects of empathy in both personal and professional life, in medical students.

Furthermore, we examined differences in empathy scores by gender, class year, and carrier intention, love for animals, interest in medical ethics, belief in God, and having an ill person in the family. For the most part, the finding of this study supported the findings of previous literature. We found clear gender differences in empathy among the participants in the study. Moreover, the students that participated in our study demonstrated empathy decline over the years as medical studies progress. Note, however, that we found a positive correlation between empathy and factors that has not been widely explored or discussed in previous literature relative to the topic of interest, such as love for animals, interest in medical ethics, belief in God, having an ill person in the family. For the most part our findings were consistent with previous literature.

To help readers grain some deeper insight into subjects related to our findings, we attempted to present below a comprehensive summary of previous research on these topics.

In this study we found a statistically significant important empathy decline among medical students over the course of medical school. This finding is consistent with previous literature. Research has shown a striking decline in empathy over the years as medical studies progress [9, 49, 56, 83–87]. More specifically, several studies have shown a steep decrease in empathy among students during their third (clinical) year of medical school, namely, as they begin their clinical training and hence empathic communication is critical [29, 88, 89]. Mirani, Shaikh, Tahir found that empathy levels were “highest in first-year students and lowest in final-year students” [90]. Importantly, it is suggested that general empathy declines less than clinical empathy [91]. As the decline in empathy among medical students over the course of medical school is a significant problem for medical education and needs to be addressed, we provide further discussion on this topic in the context of previous literature.

The pattern of decline in empathy as medical studies progress may be different for males and females or for the various components (dimensions) of empathy as well. To cite just one example, Quince et al. demonstrated no change in the cognitive aspect of empathy during the studies (neither for men nor for women). Furthermore, women’s affective empathy demonstrated no change, while men's affective empathy declined slightly during the studies [92]. The decline in empathy during medical studies may be viewed as emotional neutralisation, namely, cynicism [51]. Moreover, note that there may be conflicting patterns in empathy change demonstrated by studies conducted within the same region or between wider geo‐sociocultural regions (i.e., between the West and the Far East in the U.S.). A probable explanation for this phenomenon may be an interplay between the general and specific aspects of empathy [36]. At any rate it should be stated that while the decreasing trend of student’s empathy over the course of medical school is a multi-factorial and complex process [32], it “is not as indiscriminate (patternless) as once thought” [36].

Studies failed to support the hypothesis of a strong and generalisable trend of decline in empathy over the course of medical school [23, 36, 53, 84, 93–97] or found only declines in some aspects of empathy [98]. Surprisingly, authors reported an increase in (clinical) empathy during medical studies [99]. Handford et al. found an increase in the behavioural component of empathy [98]. Interestingly, Chatterjee et al. found that empathy “fell from the first to the third semester, then more or less plateaued, and then rose again in the seventh semester” [43]. Interestingly, it has been argued that this decline may be due to differences in methodology used in research. Note that relative studies are based largely on studies using self‐administered tools [23]. The decline in empathy may reflect self-representation changes [87]. Importantly, Ferreira-Valente et al. who contested the reliability of the decline of empathy as medical students begin their clinical training, conducted a review and concluded that the decline in empathy may be due to differences across the conducted studies in design, tools used and sample sizes [100]. The authors identified differences between cross-sectional and longitudinal studies [100]. The fact that physicians often are fully aware of the behavioral changes in themselves and their colleagues [49], may affect study results.

Culture and the educational background of students at admission may affect the trajectory of empathy levels across the years of medical education. While the decline of empathy levels as medical students progress has been noticed in many studies conducted in the United States, Europe or China, this was not the case with studies conducted in other contexts (i.e. Japan, Iran, Ethiopia, Portugal). This may be due to different cultural contexts or students’ educational backgrounds at admission [17, 36]. Note, however, that in a study, Pakistani medical students showed lower levels of empathy as compared to medical students in Western countries [101]. Empathy is a locally construed global construct [36].

Several and complex factors may drive or tackle the decrease of empathy trajectory during medical studies. Understanding the drivers of empathy decline is an important but difficult task. Not surprisingly, “determining with certainty whether it is more likely to change or to remain stable throughout medical studies has proven to be inconclusive” [102]. The lower the students’ empathic ability at the beginning of their studies the stronger the decline of their empathy levels during their studies [56, 102]. Students with high empathy levels may show not decreasing but stable trajectory during their studies [102]. Moreover, the students’ personality in the entry year may play a crucial role in forming the empathy trajectory during their studies [102].

Studies have shown relationship between medical students' personality and empathy [103–106]. Furthermore, it is suggested that medical students’ attachment styles predict empathy dimensions” [107].

Medical students’ altruistic motives for studying medicine when entering medical school have been associated with greater empathy [108, 109]. The promotion of altruistic and interpersonally oriented motives may tackle the decrease of empathy trajectory during medical studies [102]. Empathy is considered a “motivated phenomenon” “in which people may choose to experience or avoid the process of understanding other people’s emotions” [110–112].

Workplace stress and fatigue secondary to workload and increasing levels of clinical responsibility are cited in literature as factors promoting empathy decline among physicians [49]. Furthermore, “sleep loss has been shown to affect some aspects of empathy” [113]. It has been argued that the decline of medical students’ empathy during their studies may be a self-protective or coping mechanism at times of transition [23, 114–116]. However, Triffaux, Tisseron, Nasello asked whether the decline of empathy among medical students is useful coping process or dehumanization [87]. Furthermore, the fact that medical curriculum focuses more mechanistic view on illness (that may reduce the patients to a disease or an object) than humanistic values may tackle empathy decline during medical studies [5]. It is arguably said that “the more doctors depend solely on technology, the more they lose their humanism” [117]. This assumption has been supported by a systematic review and meta-synthesis of qualitative studies [118]. Further research is required to further explore the cited in the literature factors and identify factors that contribute to changes in empathy [117].

In conclusion, given that the decline of empathy during medical studies is a complex phenomenon, further studies should be conducted in larger samples of medical students to enforce the findings of this study.

As presented in “Results” section above, we found no statistically significant correlation between empathy and specialty interest among participants in this study. It is widely argued that empathy drives students to select specific specialties. Students preferring technology-oriented specialties (i.e., radiology, pathology, surgery) have lower empathy levels while students preferring people-oriented specialties (i.e., family medicine, pediatrics, internal medicine, psychiatry) have higher empathy levels [5, 9, 49, 56, 83, 87, 88, 94, 96, 104, 119]. Note, however, that some studies did not support the hypothesis of relationship between empathy specialty preference [120, 121]. Furthermore, in this study, we explored the students’ carrier intention, namely, whether they were interested in practicing medicine in the public or in the private sector, or even following academic carrier. However, we found no statistically significant correlation between empathy and carrier interest among participants in this study.

Moreover, in this study female medical students showed significantly higher empathy scores than male medical students on the Per-Cognitive, Per-Emo and Pro-Emo subscales. The empathy gender bias is a strong effect observed in several empathy-related phenomena [87]. Longitudinal and cross-sectional research has consistently associated the empathy of medical students with gender [9, 43, 83, 100, 122, 123]. Many studies have reported gender differences with females scoring higher or significantly higher than men [2, 9, 56, 88, 90, 92, 96, 99, 104, 106, 124–126]. In many studies male students showed a more significant empathy decline than females during the studies. For instance, Raof and Yassin [119] found that female students had significantly higher empathy levels than male students who showed a significant empathy decline during the studies. Shashikumar et al. did not found significant difference among female students in different semesters. However, they found significant decline in empathy among male students [85]. Ye et al. found no significant gender differences in the empathy scores among medical students before early clinical contact. Note, however, that authors reported no empathy gender differences [59]. Di Lillo et al. [127] found that although females had slightly higher empathy levels, the difference was not statistically significant. In the same vein were Iranian studies [95, 128]. The study of Tariq, Rasheed, Tavakol with Pakistani students did not support the hypothesis that female medical students have higher empathy levels than males [101]. Cultural context may be a factor affecting the gender differences in the empathy scores among medical students. As presented in “Results” section above, we found statistically significant empathy gender differences among participants in our study.

Importantly, it is arguable that further research is necessary to explore the relationship between gender and clinical empathy levels [43]. Christov-Moore et al. [1] state that further investigation of sex/gender differences in empathetic skill may be informative for understanding the nature of empathy. Moreover, it is argued that “gender differences in response to medical humanities programs require further study” [6].

Difference by gender may be more evident for some components of empathy. Quince et al. found statistically significant gender differences which however were not same in affective and cognitive empathy. As regards affective empathy, gender differences were found in all six years while cognitive empathy gender differences were found for four of the six years [92]. Many studies argued that the difference by gender is “more evident for the caring component than the sharing component” [44]. To cite just one example, Ishikawa et al. found that “female students manage to maintain their patient-centered attitude while presumably going through comparable experiences in their medical training” [44].

Among the reasons cited in the literature for the empathy gender differences are included: traditional gender roles [43], intra- and inter- cultural gender-related personality characteristics [53], the fact that self-reporting tools used in research methodology may induce biases in favor of gender-based stereotypes [129], or even “phylogenetic and ontogenetic roots” [1]. Shashikumar et al. [85] stated that female students are by nature more caring and loving. Christov-Moore et al. [1] state that “there is also a small but growing literature on gender differences in the ability to recognize *emotional body language*” and that “females are faster and more accurate than males in recognizing facial expressions”. Note that the understanding of the mechanisms underlying different subtypes of empathy is increasing [130]. Some studies highlighted the gender-related differences between the neurobiological underpinnings of empathy [1]. Schulte-Rüther et al. suggested that “females recruit areas containing mirror neurons to a higher degree than males during both SELF- and OTHER-related processing in empathic face-to-face interactions. This may underlie facilitated emotional "contagion" in females” [131]. Note, however, that in the neuroscience of empathy there may be methodological and conceptual pitfalls [132]. It is not clear whether or not there is significant difference in the activation of the various brain structures between men and women.

Last, one must bear in mind that empathy gender differences may be due to the method used in the research. Some authors suggested that, when a self-report method is used to assess empathy, women’s gender-role model is activated and hence they respond more empathically [133]. It is suggested that differences in general emotional responsiveness may be the reason for gender differences in self-reported empathy [134].

In conclusion, the prevailing trend in literature is that female medical students show higher levels of empathy than male medical students. Note, however, that this trend has been challenged by a number of scholars. Last, it should be highlighted that in the socio-demographic questionnaire that we used in this study, there was the answer option “other gender” in addition to the answer options “male” and “female”.

Furthermore, it is noteworthy that in this study we found a (statistically significant) positive correlation between trust in God and empathy among participants in our study. Empathy and religiosity seem to be linked as both underline values such as altruism, sympathy, helping and caring for others. Málaga, Gayoso and Vásquez found higher levels of empathy according to religious beliefs among medical students (“practicing a religious denomination is related to a higher level of empathy”) [126].

However, there is not a clear positive correlation between religiosity and empathy in the literature. Damiano, DiLalla et al. found that “spirituality openness was related to empathy only in nondepressed students. Empathy of students with higher levels of depression was generally lower and not affected by spirituality openness” [135]. Empathy may have a mediating role between emotional intelligence and religiosity [136]. Damiano et al. found “that meaning of life and previous mental health treatment but not Religiosity were positively related to empathy” [137]. At any rate, the relationship between religiosity and empathy needs further research.

Besides, in this study, we found no statistically significant correlation between participants’ age and empathy among participants in our study. Studies have shown a negative association between age and (cognitive) empathy. For instance, it is argued that a large subset of elderly people experience decline in their social understanding abilities with advancing years [138]. Happé, Winner, and Brownell reported that “although performance on tasks with nonmental content may decrease with age, performance on theory of mind tasks remains intact and may even improve over the later adult years” [139]. Yet, in direct contrast to this finding, Maylor et al. [140] suggested a decline in theory of mind abilities with age. Studies have found reduced neural responses in brain activity associated to empathy in older adults [141, 142]. Chen et al. [141] found that “the neural response associated with emotional empathy lessened with age, whereas the response to perceived agency is preserved”. Many studies have suggested that while cognitive empathy declines with advancing age, there is no age-related difference in affective empathy [143]. As the vast majority of the participants in this study were fallen into a narrow range of young ages, our finding were not indicative of the correlation between the variables age and empathy.

## Limitations

Some limitations on the use of a self-reported questionnaire to measure empathy can be raised. Braun et al. stated: “For more than 30 years, the interpersonal reactivity index (IRI) has been used to measure the multidimensional aspects of empathy” [144]. First, data identified by self‐reported empathy instruments may involve lack of accuracy and biases causing respondents to adopt traditional gender-based stereotypes [73, 129]. Second, as empathy is a psychosocial phenomenon, qualitative studies play a considerable role in providing further insight into empathy and capture the complexities of human experience concerning empathy [73]. Third, it is arguably stated that perspective-taking subscales may support the understanding of another's emotions rather than the understanding of another's perspective [31]. It is argued that “a discrepancy exists between self-administered empathy scores and observed empathic behaviours” [23]. As medical students acculturate to illness and suffering through real‐world experience, their behavioural skills may evolve, thus developing a trend towards better patient‐centred communication [42]. Therefore, the decline of empathy on a self‐administered tool, may “not sufficiently predict actual empathic behaviour towards patients” [23].

Furthermore, this study is a cross-sectional study. In literature, both cross-sectional and longitudinal studies have been conducted to explore the phenomenon empathy among medical students. Moreover, this study has used group-level method. This is another limitation of this study. It is true that the vast majority of longitudinal studies on medical student empathy have used group-level methods which, however, cannot assess inter-individual changes (namely, differences between individuals) and intra-individual changes (namely, differences within the same person over time) [100, 102]. “An understanding of empathy would be incomplete without a consideration of individual differences” [1].

Finally, and most importantly, in this study, an increased number of missing values was obtained for Professional setting. A significant negative correlation was found between the number of missing replies of each participant and the study year (Pearson =  − 0.336, *p*. < 0.000), with students from 1rst and 2nd years mainly not responding to most items.

## Conclusions

The preliminary validation of the 52-item Greek version of the Toronto Composite Empathy Scale (TCES) demonstrated acceptable validity and reliability among medical students and could be further tested in larger samples of medical students. The TCES might be considered as a psychometrically sound instrument for measuring the cognitive and emotional aspects of empathy, in both personal and professional life, in medical students. The overall reliability analysis of the TCES questionnaire was found to be high (Cronbach's α = 0.895, *p*. from Hotelling’s T-Squared Test < 0.000). We assessed the convergent and divergent validity of the Scale. Furthermore, we found a statistically significant difference between the levels of cognitive and emotional empathy in both personal and clinical settings. The levels of emotional empathy were lower than the levels of cognitive empathy, with the levels of professional emotional empathy being the lowest.

The mean total score of empathy showed that students had a moderately high empathy. The 52‐item TCES, 26 for the personal (Per) setting and another 26 for professional (Pro) life, equally divided into cognitive (Cog) and emotional (Emo) empathy in each case. It was found that there is a statistically significant difference in means between the Per-Cog and Per-Emo settings (*p* < 0.001), the Pro-Cog and Pro-Emo (*p* < 0.001), the Per-Cog and Pro-Cog (*p* = 0.004), and the Per-Emo and Pro-Emo (*p* < 0.001). Females had significantly higher empathy scores (mean score 208.04) than males (mean score 192.5) on the Per-Cognitive, Per-Emo and Pro-Emo subscales. Furthermore, a positive correlation was found between empathy and factors such as love for animals, interest in medical ethics, belief in God, having an ill person in the family, class year or carrier intention.

For the most part our findings were consistent with previous literature. However, we identified some nuances that might draw researchers’ attention. The results of this study may contribute to design appropriate educational strategies and interventions in the curriculum to enhance empathy in the medical students.

## Supplementary Information

**Additional file 1.** Supplementary Tables.

## Data Availability

The authors declare that the data analyzed during the current study and supporting the findings of this study are available from the corresponding author on reasonable request. All data generated or analyzed during this study are included in this published article.

## Abbreviations

TCES: Toronto composite empathy scale
GMC: General Medical Council
AAMC: Association of American Medical Colleges
PAM: Perception–action model
IRI: Interpersonal reactivity index
SPSS: Statistical product and service solutions
IBM: International Business Machines Corporation
SD: Standard deviation
ANOVA: Analysis of variance
JSE: Jefferson scale of empathy
IRI: Interpersonal reactivity index
EQ: Empathy quotient
E-Scale: Empathy scale
