# Supplementary material for: Reliability of Greek version of the Toronto empathy questionnaire in medical students and associations with sociodemographic and lifestyle factors
Source: BMC Psychol. 2022 May 2;10:113. doi: 10.1186/s40359-022-00824-6 (PMC9063083; doi:10.1186/s40359-022-00824-6)
Supplement: Supplementary file 1 — Additional file 1. Supplementary Tables. [file 40359_2022_824_MOESM1_ESM.docx]

**Additional File**

Supplementary Table 1.

The effect of gender and years of studies on total score.

| **Descriptive Statistics** | | | | |
| --- | --- | --- | --- | --- |
| Dependent Variable: total_score | | | | |
| GENDER | Study_year | Mean | Std. Deviation | N |
| Male | 1rst year | 195.1818 | 32.88409 | 11 |
|  | 2nd year | 194.9000 | 11.37688 | 10 |
|  | 3rd year | 203.8333 | 11.65190 | 6 |
|  | 4rth year | 193.5000 | 26.66646 | 6 |
|  | 5th year | 192.5714 | 25.88344 | 7 |
|  | 6th year | 186.0000 | 16.23128 | 12 |
|  | degree | 167.5000 | 20.50610 | 2 |
|  | Total | 192.5000 | 22.24287 | 54 |
| Female | 1rst year | 205.9615 | 25.43931 | 26 |
|  | 2nd year | 208.0000 | 13.88968 | 14 |
|  | 3rd year | 206.7143 | 23.80921 | 28 |
|  | 4rth year | 207.7500 | 25.09050 | 16 |
|  | 5th year | 206.8000 | 29.47202 | 15 |
|  | 6th year | 213.1667 | 20.69171 | 18 |
|  | degree | 231.0000 | . | 1 |
|  | Total | 208.0424 | 23.39932 | 118 |
| Total | 1rst year | 202.7568 | 27.83424 | 37 |
|  | 2nd year | 202.5417 | 14.25537 | 24 |
|  | 3rd year | 206.2059 | 22.03686 | 34 |
|  | 4rth year | 203.8636 | 25.71325 | 22 |
|  | 5th year | 202.2727 | 28.57435 | 22 |
|  | 6th year | 202.3000 | 23.11165 | 30 |
|  | degree | 188.6667 | 39.42503 | 3 |
|  | Total | 203.1628 | 24.08943 | 172 |

Supplementary Table 2.

| **Tests of Between-Subjects Effects** | | | | | | |
| --- | --- | --- | --- | --- | --- | --- |
| Dependent Variable: total_score | | | | | | |
| Source | Type III Sum of Squares | df | Mean Square | F | Sig. | Partial Eta Squared |
| Corrected Model | 12805.782^a^ | 13 | 985.060 | 1.801 | .047 | .129 |
| Intercept | 3065876.000 | 1 | 3065876.000 | 5604.914 | .000 | .973 |
| GENDER | 8248.942 | 1 | 8248.942 | 15.080 | .000 | .087 |
| Study_year | 472.656 | 6 | 78.776 | .144 | .990 | .005 |
| GENDER * Study_year | 3521.225 | 6 | 586.871 | 1.073 | .381 | .039 |
| Error | 86425.660 | 158 | 546.998 |  |  |  |
| Total | 7198552.000 | 172 |  |  |  |  |
| Corrected Total | 99231.442 | 171 |  |  |  |  |
| a. R Squared = .129 (Adjusted R Squared = .057) | | | | | | |

Supplementary Table 3. Statistically significant mean differences in the subscales based on demographic characteristics.

| Subscale | Demographic | Mean | Sig |
| --- | --- | --- | --- |
| Prof-Cog | In my family there was or is a person who is seriously ill | 56.65 | 0.019 |
|  | No ill person | 59.61 |  |
| Per-Cog | I love animals: very much | 58.62 | 0.001 |
|  | I love animals: quite | 56.58 |  |
|  | I love animals: a little | 53.53 |  |
|  | I love animals: I do not care about animals | 52.5 |  |
|  | I love animals: I despise them | - |  |
| Per-Emo | I love animals: very much | 46.05 | 0.019 |
|  | I love animals: quite | 45.24 |  |
|  | I love animals: a little | 49.30 |  |
|  | I love animals: I do not care about animals | 47.67 |  |
|  | I love animals: I despise them |  |  |
| Prof-Cog | I love animals: very much | 59.30 | 0.000 |
|  | I love animals: quite | 58.44 |  |
|  | I love animals: a little | 54.46 |  |
|  | I love animals: I do not care about animals | 56.33 |  |
|  | I love animals: I despise them | - |  |
| Prof-Emo | I love animals: very much | 41.86 | 0.023 |
|  | I love animals: quite | 41.03 |  |
|  | I love animals: a little | 43.15 |  |
|  | I love animals: I do not care about animals | 43.33 |  |
|  | I love animals: I despise them | - |  |
| Pro-Cog | 1rst year of studies | 60.71 | 0.004 |
|  | 2nd year of studies | 57.64 |  |
|  | 3rd year of studies | 57.03 |  |
|  | 4th year of studies | 57 |  |
|  | 5th year of studies | 60.34 |  |
|  | 6th year of studies | 58.1 |  |
|  | Degree | 43 |  |
| Prof-Cog | Academic | 56.63 | 0.048 |
|  | Practice medicine in the public sector | 56.33 |  |
|  | Practice medicine in the private sector | 61.37 |  |
|  | Not decided | 58.18 |  |
| Prof-Emo | Academic | 37.27 | 0.027 |
|  | Practice medicine in the public sector | 44 |  |
|  | Practice medicine in the private sector | 39.25 |  |
|  | Not decided | 42.27 |  |
| Pro-Cog | Believe in a God: yes | 58.31 | 0.023 |
|  | Believe in a God: no | 55.37 |  |
|  | Believe in a God: Do not know/do not answer | 61.07 |  |
| Per-Cog | Interest in medical ethics: very much | 59.56 | 0.000 |
|  | Interest in medical ethics: enough | 56.60 |  |
|  | Interest in medical ethics: a little | 52.13 |  |
|  | Interest in medical ethics: not at all | 51.5 |  |
| Per-Emo | Interest in medical ethics: very much | 46.79 | 0.000 |
|  | Interest in medical ethics: enough | 47.03 |  |
|  | Interest in medical ethics: a little | 40.68 |  |
|  | Interest in medical ethics: not at all | 33.5 |  |
| Pro-Cog | Interest in medical ethics: very much | 60.34 | 0.007 |
|  | Interest in medical ethics: enough | 57.96 |  |
|  | Interest in medical ethics: a little | 53.86 |  |
|  | Interest in medical ethics: not at all | 56 |  |
| Pro-Emo | Interest in medical ethics: very much | 42.74 | 0.000 |
|  | Interest in medical ethics: enough | 42.64 |  |
|  | Interest in medical ethics: a little | 35.27 |  |
|  | Interest in medical ethics: not at all | 29.5 |  |

Supplementary Table 4. Mean (SD) of the TCES by demographic characteristics, Sig. from Independent Sample T-test or One-Way ANOVA for equality of means.

| Demographics | Mean (SD) | Sig |
| --- | --- | --- |
| Male | 192.5 (22.24) | 0.000 |
| Female | 208.04 (23.39) |  |
| I love animals: very much | 205.84 (23.70) | 0.001 |
| I love animals: quite | 201.32 (21.83) |  |
| I love animals: a little | 200.46 (24.31) |  |
| I love animals: I do not care about animals | 199.83 (23.27) |  |
| I love animals: I despise them | - |  |
| Believe in a God: yes | 204.92 (23.43) | 0.014 |
| Believe in a God: no | 192.79 (24.28) |  |
| Believe in a God: Do not know/do not answer | 209 (23.59) |  |
| Interest in medical ethics: very much | 209.56 (22.28) | 0.000 |
| Interest in medical ethics: enough | 204.24 (21.99) |  |
| Interest in medical ethics: a little | 181.95 (24.60) |  |
| Interest in medical ethics: not at all | 170.50 (3.53) |  |

Supplementary Table 5. Statistical difference in mean total score of two specialty categories, A (people-oriented specialties) and T (technology-oriented specialties)

Descriptive statistics for the variable specialty category.

| **Specialty** | | | | | |
| --- | --- | --- | --- | --- | --- |
|  | | Frequency | Percent | Valid Percent | Cumulative Percent |
| Valid | technology-oriented specialty | 62 | 27.7 | 47.0 | 47.0 |
|  | people-oriented specialty | 70 | 31.3 | 53.0 | 100.0 |
|  | Total | 132 | 58.9 | 100.0 |  |
| Missing | System | 92 | 41.1 |  |  |
| Total | | 224 | 100.0 |  |  |

Supplementary Table 6.

Subgroups of the following 17 demographics did not have statistically significant total mean score differences:

1 – Age group

2 – Place being born (city, village etc)

3 – Place grown (city, village etc)

4 – Born in Greece or not

5 – Grown in Greece or not

6 – Grown in family with few or many members

7 – Living alone or with others

8 – Having siblings

9 – Having children

10 – Grown with grandparents or other elderly people

11 – In my family or close environment there is a person seriously ill

12 – Having or had a serious health problem

13 – Financial status of family in which has grown

14 – Having parents with a Bachelor degree

15 – Year of studies

16 – Specialty that intent to choose (53 categories)

17 – Intention to work (in academia, public sector, private sector, do not know yet)
